# Sub-terahertz transmissive reconfigurable intelligent surface for integrated beam steering and self-OOK-modulation

**DOI:** 10.1038/s41377-024-01690-0

**Published:** 2025-01-01

**Authors:** Dongfang Shen, Feng Lan, Luyang Wang, Tianyang Song, Munan Yang, Tianyu Hu, Yueting Li, Xiaolei Nie, Jiayao Yang, Shixiong Liang, Hongxin Zeng, Hui-Fang Zhang, Pinaki Mazumder, Ziqiang Yang, Yaxin Zhang, Tie Jun Cui

**Affiliations:** 1 Sichuan THz Communication Technology Engineering Research Center, School of Electronic Science and Engineering, University of Electronic Science and Technology of China, Chengdu, 611731 China; 2 Yangtze Delta Region Institute (Huzhou), University of Electronic Science and Technology of China, Huzhou, 313000 China; 3 Zhangjiang Laboratory, Shanghai, 201204 China; 4 School of Microelectronics, Tianjin University, Tianjin, 300072 China; 5 Shenzhen Institute for Advanced Study, University of Electronic Science and Technology of China, Shenzhen, 518100 China; 6 Department of Electrical Engineering and Computer Science, University of Michigan, Ann Arbor, MI 48109 USA; 7 Southeast University, Nanjing, 210096 China

**Keywords:** Metamaterials, Microresonators

## Abstract

Boasting superior flexibility in beam manipulation and a simpler framework than traditional phased arrays, terahertz metasurface-based phased arrays show great promise for 5G-A/6G communication networks. Compared with the reflective reconfigurable intelligent surface (reflective RIS), the transmissive RIS (TRIS) offers more feasibility for transceiver multiplexing systems to meet the growing demand for high-performance beam tracking in terahertz communication and radar systems. However, the terahertz TRIS encounters greater challenges in phase shift, beam efficiency, and complex circuitry. Here, we propose a sub-terahertz TRIS based on the phase shift via Pancharatnam-Berry (PB) metasurface and self-on-off keying (OOK) modulation via Schottky diodes. The electrically reconfigurable unit cell consists of a column-wise phase resonator and a rectangular slot. An experimental retrieved equivalent lumped-element circuit model is implemented in joint field-circuit simulations and is validated by experiments. A fabricated prototype demonstrates excellent performance of TRIS with the minimum insertion loss of 2.8 dB for operational states, large bandwidth nearly covering the entire W-band for 1-bit phase shift, deep OOK amplitude modulation of 12 dB, and wide scanning range of ±60° with low specular transmission. We further implement an integrated platform combining high-speed beam steering and spatial-light modulation, verifying the point-to-point signal transmissions in different directions using the TRIS platform. The proposed TRIS with high-performance and cost-effective fabrication makes it a promising solution to terahertz minimalist communication systems, radar, and satellite communication systems.

## Introduction

Due to the prohibitive cost and complexities associated with traditional phased arrays^1^, terahertz (THz) reconfigurable intelligent surface (RIS) has emerged as a leading technology to address the persistent challenge of beam tracking for 6G communications^2–4^. By integrating active components within subwavelength-scaled unit cells, RIS systems offer versatile beamforming capabilities. These systems are capable of managing multi-dimensional electromagnetic modulations, including phase, amplitude, polarization, and harmonic waves, in intricate communication settings^5–7^.

Recent advances in the field have seen many successful implementations of reflective RIS technologies. These implementations are driving forward systematic applications within the terahertz spectrum^8–15^, Notable developments include a 0.235 THz high electron mobility transistor (HEMT) -based array, the largest of its kind, measuring 98 × 98 by HRL Laboratory^8,9^, a 0.265 THz Complementary metal-oxide-semiconductor transistor (CMOS) -based 32 × 32 array developed by MIT and Intel^10^, and a 0.34 THz HEMT-based 64 × 64 array by UESTC^11^. In contrast, the feasibility of integrating TRIS with the transmitters is greater. It can reduce the path loss caused by spatial feeding and further achieve the miniaturization of RIS systems^16^. However, advancements in terahertz transmissive RIS (TRIS) are progressing more slowly due to significant challenges in achieving both high transmittance and substantial phase shifts. In 2020, Princeton University reported a programmable 24 × 24 CMOS array of transmissive two-dimensional beamforming intelligent metasurfaces, capable of single-beam scanning within a ± 30° range at a frequency of 0.3 THz^17^. That same year, Tsinghua University introduced a geometric phase transmission metasurface based on diodes, featuring a 32 × 2 array with a one-dimensional scanning angle range of ±30°^18^. Further developments in 2021 by Southeast University reported a liquid crystal-based 1-bit 48 × 48 array transmissive metasurface, achieving a ±30° one-dimensional beam scanning at 0.426 THz^19^. Nevertheless, in higher frequency bands beyond the W-band and sub-terahertz band, TRIS development faces challenges. These challenges include high switch parasitics, limited phase shifts, low transmittance, and significant interference from multi-layered structure feedlines. Addressing these challenges necessitates urgent research into de-embedding modeling methods, innovative phase shift structures, and low-interference feeding architectures.

Recent research has focused on advancing information modulation techniques using RIS to create streamlined and secure communication systems. Microwave-band transmitters utilizing TRIS technology, such as binary frequency shift keying (BFSK)^20^, quadrature phase shift keying (QPSK)^21^, 256 quadrature amplitude modulation (256-QAM)^22^, and multi-channel transmitters employing spatiotemporal coding^23^, have been explored. Furthermore, terahertz transmitters that use TRIS technologies are proving valuable for communication systems. They help streamline these systems, enhance signal stability, and reduce interference. This is achieved through the integration of high-speed beam steering and information modulation. However, significant challenges remain in implementing multi-channel transmitters with spatiotemporal modulations in the terahertz or sub-terahertz bands^24–26^. These challenges are primarily due to limitations in-phase-shift performance and tuning efficiency, which lead to reduced radiation efficiency and noticeable path losses.

This study introduces an innovative sub-terahertz TRIS that utilizes Schottky diodes for controlled on-off keying (OOK) PB-phase (OOK-PB-phase) on coding metasurfaces. This meta-device switches between two transmission states by adjusting external voltages to manipulate the states of the Schottky diodes, enabling 180° phase modulation with the cross-polarization component based on the generalized PB phase principle. Demonstrated through an 8 × 8 OOK-PB-phase TRIS array developed using printed circuit board (PCB) technology, this device exhibits a remarkable cross-polarization transmittance of −2.8 dB at 100 GHz, with a relative bandwidth of −3 dB covering 11%. It maintains a phase shift of 180° ± 10° throughout its operational frequency spectrum, thus facilitating independent control over both amplitude and phase. Moreover, it achieves a maximum angular scanning range of ±60°, effectively minimizing specular reflections. Our research introduces a trailblazing wireless communication paradigm using TRIS, incorporating simultaneous adjustments in the beam angle and real-time OOK modulation across the frequency range of 97.5–107.5 GHz. This allows for the development of an integrated platform combining high-speed beam steering and spatial light modulation, thereby reducing system complexity and insertion loss while potentially enhancing transmit power. Our proposed TRIS prototype offers a cost-effective, high-performance solution for terahertz joint sensing and communication (JSAC) systems, RIS-assisted 6G base stations, imaging, and communication systems.

## Results

### Numerical analysis and discussion

**Fig. 1:**
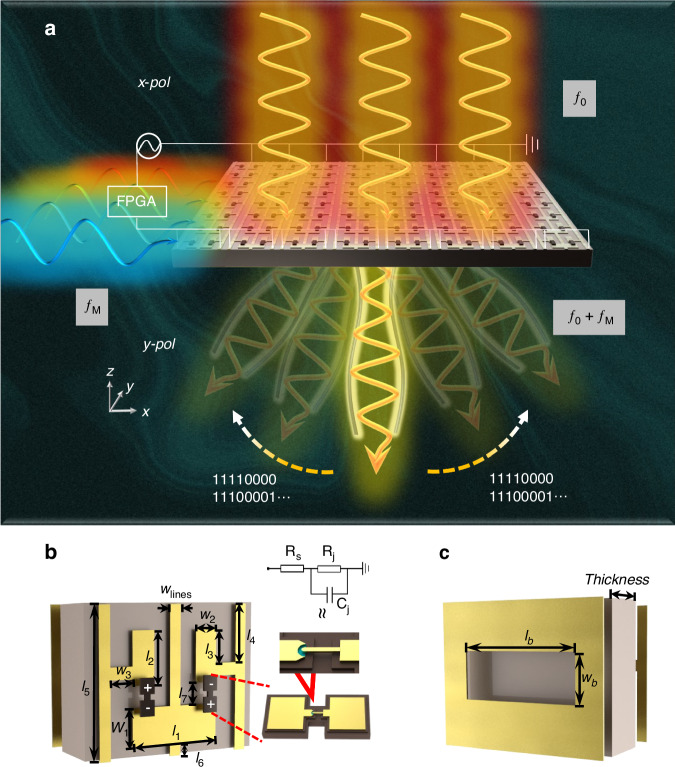
**The 3D schematic of the designed TRIS array and unit structure.** **a** The TRIS is capable of simultaneously transmitting beam angle deviation and OOK modulation of signals. **b** This illustrates the top view of the TRIS unit structure. **c** This illustrates the bottom view of the TRIS unit structure

The conceptual schematic of the proposed TRIS is depicted in Fig. 1a. When powered by an additional voltage source, the TRIS can switch between various coding sequences to create different phase gradients. This allows for deflecting incident carrier waves of frequency *f*_0_ into desired transmissive beams in one dimension. Furthermore, in addition to its primary beam steering function, TRIS can also achieve a self-OOK modulation function. This modulation mode involves a voltage source modulated at frequency *f*_M_, enabling simultaneous beam steering and amplitude modulation. In Fig. 1b, c, the unit cell of the TRIS comprises a dual-split E-shaped resonator on the top and a rectangular slot at the bottom. These are separated by a TSM-DS3 dielectric layer (with *ε*_*r*_ = 3, $tan\delta$ = 0.0011, and a thickness of 500 μm). The dual-split E-shaped resonator is positioned between two parallel outstretching anode wires, with a central wire functioning as the common ground. Additionally, two low-barrier Schottky diodes are placed with opposite polarity at the splitting gaps of the E-shaped structure. By controlling the cutoff and conductive states of these diodes on the left and right sides of the resonator, the TRIS’s operation can be regulated. The rectangular slot acts as a polarization selector for cross-polarized components from the E-shaped resonator. From the perspective of transmissive polarization conversion, the high polarization conversion efficiency of the meta-atom structure can be understood through multiple interference theories^27^. This improves the cross-polarization transmission efficiency. (The rationale for the bottom slot is described in Supplementary Fig. S8) The unit cell’s dimensions are 1600 μm × 1200 μm, approximately half of the wavelength at 94 GHz along the *x* direction, allowing accommodation for the two relatively large-sized diodes. (Details of the meta-atom structure parameters are described in Supplementary Fig. S1) All structural parameters are tailored to meet PCB processing limitations^28,29^, such as a minimum line width of 100 μm and a precision of 10 μm. The metallic patterns are composed of copper film plated with gold, featuring a thickness of 18 μm (0.5 oz). (Details of the PCB process limitations in Supplementary Fig. S2)

A combined field-circuit approach is employed to model the unit cell in CST Studio Suite, incorporating the three-dimensional diode structure and its R-C lumped circuitry from the Advanced Design System (ADS). This method aims to explore the amplitude and phase modulation characteristics of the unit cell. By utilizing S-parameter measurements on the unit-cell loaded waveguide, the equivalent circuit parameters of the diode are extracted through the reverse fitting of the measurement results to the ADS lumped-circuit simulation outcomes. The extracted equivalent lumped parameters of the diode include *R*_s_ = 1 ohm, *C*_j_ = 4 fF, *R*_joff_ = 983 ohm, and *R*_jon_ = 0.7 ohm.

**Fig. 2:**
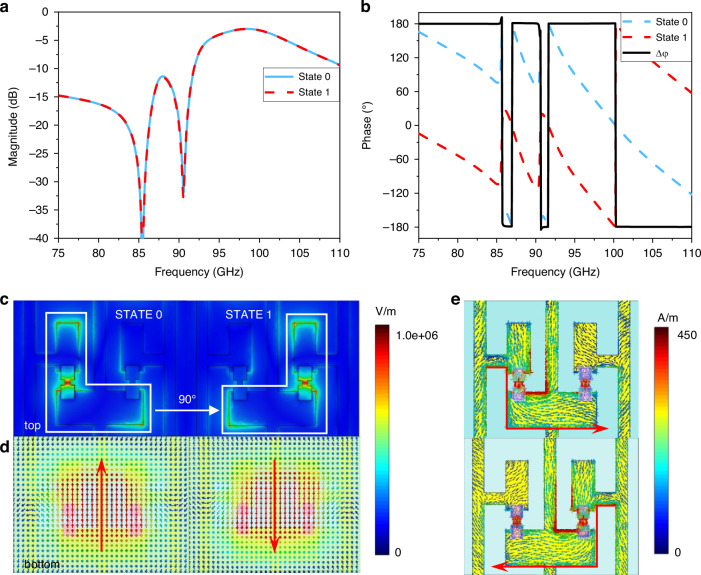
**The numerical simulation results of TRIS.** **a** and **b** depict the variation in amplitude, phase, and phase shift of TRIS when the Schottky diodes control the on-off states, denoted as STATE 0 and STATE 1, respectively. **c** The electric field distribution of the top layer, **d** the electric field vector distribution of the bottom layer, and **e** the surface current vector distribution of STATE 0 and STATE 1 at 99.5 GHz, respectively

Following the principle of generalized PB phase, the rotation angle *θ* and the generalized geometric phase shift *ф* of the unit can be approximated as^30^:

1 $$\phi =\left\{\begin{array}{ll}\pm\!2n\theta , & n\,{\rm{is}}\,{\rm{odd}}\\ \pm\ \!n\theta , & n\,{\rm{is}}\,{\rm{even}}\end{array}\right.$$

where *n* denotes the order of rotational symmetry, and the ± sign is determined by the rotation direction of the principal axis. Due to the one-fold rotational symmetry (*C*_1_) of the dual-split E-shaped resonator, a geometric phase of ±2*θ* can be generated by mimicking the rotation of the L-shaped structures that make up the E-shaped resonator, as illustrated in Fig. 2c. This bias-controlled rotational resonator achieves a 1-bit differential phase shift (0°/180°) between “STATE 0” and “STATE 1” by maintaining a 90° rotation concerning the reversed surface current in the equivalent geometric structure, as discussed subsequently. The states, “STATE 0” and “STATE 1”, are defined such that the Schottky diode at the left junction is conductive in “STATE 0”, while the diode at the right junction is off, and vice versa for “STATE 1”. Analysis using a frequency domain solver in CST Studio Suite under normal incidence with x-polarization reveals an overlapped cross-polarized transmission coefficient peak at 99.5 GHz with −2.5 dB for the two states, resulting in a 14% fractional bandwidth (93–107 GHz) over −3 dB, as depicted in Fig. 2a. As shown in Fig. 2b, the 1-bit phase difference achieves a smooth range of 180 ± 10° covering the entire frequency span of the W-band (75–110 GHz). Thanks to the well-matched design of the PB-phase resonator and the precisely modeled diodes, the OOK-PB-phase TRIS features low insertion loss of less than −3 dB and broadband 1-bit phase shift control, ensuring the subsequent integration of beam steering and self-OOK modulation.

To provide a more comprehensive understanding of the physical mechanism behind phase-only modulation, the distributions of the surface electric field and surface current of the unit cell under STATE 0 and STATE 1 at 99.5 GHz are illustrated in Fig. 2c, d. In Fig. 2c, the intense resonant electric fields above the two states delineate two L-shaped zones framed by white lines, which can be rotated by 90° around the central axis, simulating an equivalent mirrored unit cell. As seen in Fig. 2e, STATE 0, with the left-side diode “on” and the right-side diode “off,” triggers a left-downward electron flow within the L-shaped structure on the right side. Conversely, STATE 1, with the left-side diode “on” and the right-side diode “off,” induces a right-downward electron flow within the L-shaped structure on the left side. The surface currents under the two states, as indicated by the red arrows in Fig. 2e, flow in opposite directions with identical amplitude distributions, resulting in a 180° phase difference and consistent transmissive amplitudes. Following the PB phase principle, a 1-bit differential phase shift (0°/180°) can be achieved when the resonant L-shaped structure is rotated symmetrically by 90° between STATE 0 and STATE 1. Acting as a polarization filter, the bottom rectangular slot enhances the *x-to-y* cross-polarization conversion in transmission, as indicated by the E-plane electric field vector distributions in Fig. 2d.

### Programmable beam scanning experiment

**Fig. 3:**
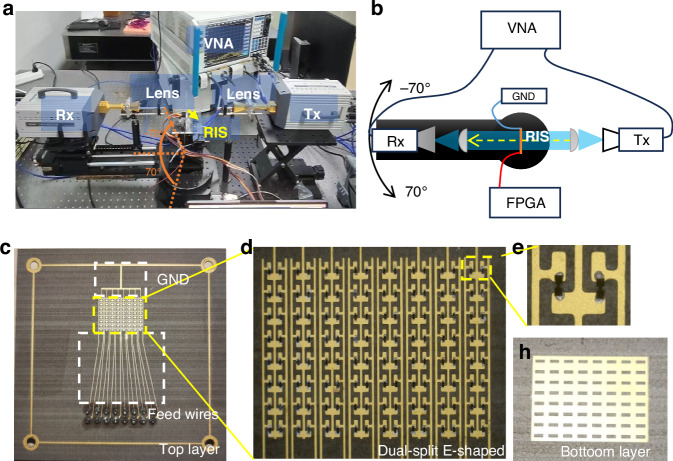
**The VNA testing setup and sample display for OOK-PB-phase TRIS.** **a**, **b** The schematic diagram of the beam measurement test platform based on the VNA system. **c** Top layer of the 8 × 8 TRIS sample. **d** Dual-split E-shaped array and **e** Unit cell under a microscope. **h** Bottom layer of the 8 × 8 TRIS sample

Based on the PCB process-oriented unit-cell design approach, a proof of concept was developed and fabricated using PCB technology, as depicted in Fig. 3c. The prototype consists of an 8 × 8 array of unit cells (12.8 × 9.6 mm², Fig. 3d,h), 128 Schottky diodes (two diodes per unit cell, Fig. 3e), and 16 pins 2.54 mm connectors, arranged in a linearly-controlled array with 8 1 × 8 unit subarrays.

The two diodes within each unit cell are aligned in the same orientation using a conductive adhesive, with the central extension wire extending to the common ground and the two positive electrodes connecting to the bias feed via gold wire jumpers between the left-side/right-side wires and the 16 pins. The measurement setup includes a sub-terahertz vector network analyzer (VNA), two collimating lenses, a Field Programmable Gate Array (FPGA) control module, 75~110 GHz T/R modules, and a rotating table, as illustrated in Fig. 3a, b. (Details of the lens collimating process and link analysis are described in the Supplementary Fig. S10)

**Fig. 4:**
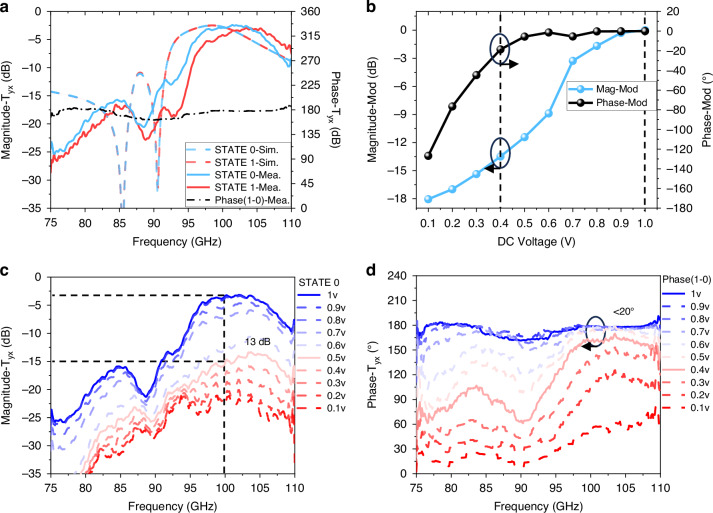
**The amplitude and phase response of OOK-PB-phase TRIS.** **a** Simulation and measurement of cross-polarization transmission amplitude response and phase difference for STATE 0 and STATE 1. **b** Modulation depth of STATE 0 and phase difference under different DC voltage feedings at 100 GHz. **c** Cross-polarization transmission amplitude response of STATE 0 and **d** Cross-polarization transmission phase difference under different DC voltage feedings

By applying a 1 V DC voltage to the Schottky diodes through the FPGA module, the amplitude and phase responses are measured under normal incidence, and compared with the simulated results shown in Fig. 4a. To account for path loss, the direct transmittance from the Tx to Rx is subtracted from the measurement results. When a 1 V DC bias is applied for phase-only modulation, the cross-polarization transmittance *T*_yx_ of STATE 0 reaches a maximum of −2.8 dB at 100 GHz, with a 1.2 dB difference compared to STATE 1. The −3 dB transmission bandwidth extends from 97.5 GHz to 107.5 GHz, resulting in a relative operational bandwidth of 11%. A continuous 180° ± 10° transmission phase difference between the two states covers the full frequency range of the W-band (75–110 GHz).

There are slight differences in amplitude and phase responses, such as a blue shift and enlarged amplitude inconsistency which may be attributed to fabrication uncertainties, including lower PCB processing accuracy, inaccurate diode modeling, and imperfect mirror symmetry of the diodes. In Fig. 4b, the joint amplitude-phase modulation depth between STATE 0 and STATE 1 is depicted under varying DC feed voltages from 0 V to 1 V (in intervals of 0.1 V) at 100 GHz, with the 1 V results serving as a reference for all bias voltages. Figure 4c illustrates the variations in cross-polarization transmittance of STATE 0, while Fig. 4d shows the changes in-phase shifts between STATE 0 and STATE 1 under different DC voltage signals.

To support the OOK communication platforms alongside beamforming capabilities, a balance between significant amplitude modulation and minimal deviation in 180° phase modulation must be achieved. Notably, for voltages of 0.4 V and above, the phase difference between the two states remains at 180° with a deviation below ±20°, meeting the requirements for OOK modulation of “0-OFF” and “1-ON” states. Additionally, the insertion loss at 0.4 V exhibits a difference of over 13 dB when compared to the 1 V bias, ensuring proper modulation for communication purposes.

According to the generalized Snell’s law, the elevation angle *θ* of the main lobe on a 1D 1-bit metasurface can be predicted as^31^:

2 $$\theta =\arcsin \left(\frac{\pm \pi +k\cdot Nd\,\sin {\theta }_{i}}{k\cdot Nd}\right)$$

where ±π represents the phase difference between the “0” and “1” states of the 1-bit encoding; d is the period of a single element, and N is the coding period, i.e., the number of adjacent in-phase coding elements. As indicated by Eq. (2), the OOK-PB-phase TRIS can digitally adjust the transmitted beam within the range of ±60° by altering the encoding sequence with changes in N. In an ideal scenario, a larger array featuring more coding phase states allows for a greater variety of N values and reduced quantization errors. However, this also leads to increased complexity in the feeding network and insertion loss, particularly for the transmit array.

**Fig. 5:**
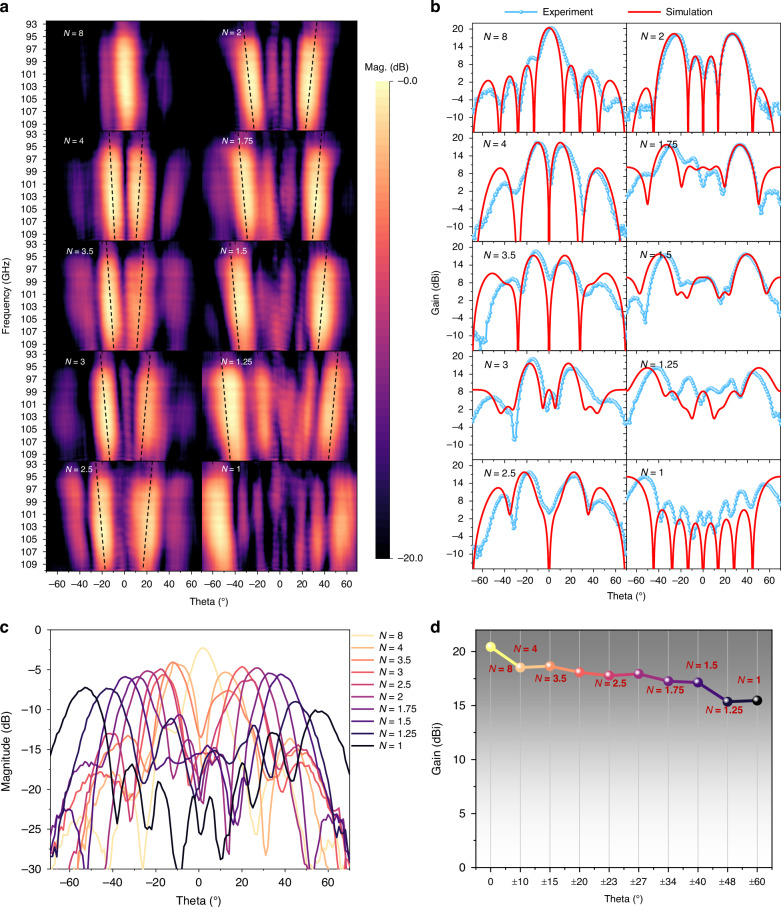
**The beam scanning experimental results for N ranging from 1 to 8.** **a** The broadband beam scanning from 97.5 GHz to 107.5 GHz, covering angles from 0° to ±60°. **b** The actual gain (blue curve) and simulated gain (red curve) results at various angles at 100 GHz. **c** The 1D amplitude results at different angles at 100 GHz. **d** The maximum gain of the main beam at different angles

To account for path loss and spillover loss, the normalized steered beam amplitude is compared with that of a hollow PCB board of the same size, used as a reference (measured in the same polarization direction). The beam angle varies with N and frequencies from 0° to ±60° in the range of 97–107 GHz in Fig. 5a, aligning well with Snell’s Law (indicated by a dotted line). At 100 GHz, the highest beam efficiencies and weakest specular transmissions are observed for all deflected cases, thanks to the ideal 1-bit amplitude-phase response displayed in Fig. 4b.

The simulated and measured beam scanning patterns at 100 GHz are visualized in Fig. 5b, c, showcasing main-lobe angles that closely match the simulated scanning angles of 0° to ±60° with an average angular error of 2°. This indicates the broad coverage of wide-angle beam scanning and the high-precision directivity of the proposed TRIS. Due to the broadband constant phase-shift response, specular transmission is effectively suppressed to below −14 dB in all coding states except *N* = 8 (at 0°). Additionally, sidelobe level (SLL) suppression exceeds 12 dB at 0° and remains above 5 dB up to ±60°.

Accounting for diffraction edge effects, the beam amplitude of the main lobe from 0° to ±60°, gradually decreases from −2.8 dB to −7 dB, with the half-power beamwidth (HPBW) expanding from 10° to 16° as depicted in Fig. 5c. This trend of main-lobe gain is similarly observed in Fig. 5d, where the main-lobe gain peaks at 20.4 dBi at 0° and decreases to 15 dBi at ±60°. Overall, the discrepancies between the simulations and measurements become more pronounced with the increasing angular deflection, likely due to nonideal phase distributions stemming from array nonuniformity and measurement errors.

### Integrated beam steering and self-OOK modulation experiment

**Fig. 6:**
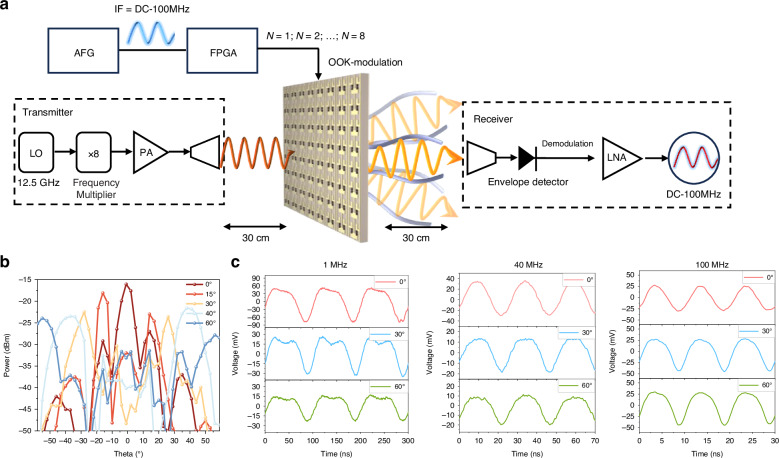
**The validation results of the wireless communication prototype.** **a** The schematic diagram of the sub-terahertz integrated platform combining high-speed beam steering and self-OOK modulation. **b** The power transmission of the 100 MHz single-tone signal at various scanning angles. **c** The demodulated signal waveforms at 0°, 30°, and 60° scanning angles for different frequencies of single-tone signals

The prototype device demonstrates the ability to jointly modulate amplitude and phase, exerting the PB-phase trait of broadband constant phase shift, an integrated platform combining high-speed beam steering and self-OOK modulation has been developed as depicted in Fig. 6a. A sinusoidal AC signal ranging from DC to 100 MHz is supplied to the bias module by the arbitrary function generator (AFG), with an absolute voltage range for AC signals set between 0.4 V and 1 V. The AFG specification is Tektronix AFG3101C with a maximum output frequency of 125 MHz. Based on the amplitude-phase response at various voltages, the 1-bit coding thresholds are set at 1 V (upper) and 0.4 V (lower). This AC bias application ensures modulation of the deflected beam at a fixed elevation angle, thereby minimizing channel interference due to phase shift misalignment caused by voltage variations.

As a final demonstration of the versatility of TRIS, we present a point-to-point tracking self-modulation transmission system (see Supplementary Video). Figure 6c illustrates the demodulated signal sinusoidal waveforms at various tracking angles for single-tone modulation rates of 1 MHz, 40 MHz, and 100 MHz. The waveform distortion is more noticeable at lower single-tone modulation rates, particularly at 1 MHz. However, as the modulation rate increases, the impact of low-frequency noise diminishes, leading to a significant reduction in distortion and a more regular waveform at 40 MHz and 100 MHz. The previous terahertz wireless communication experiments with OOK modulators have shown that Schottky diodes of the same model can achieve a response rate of 25 GHz^32^. This suggests that by addressing the noise issue in the feeding module, the OOK-PB-phase TRIS could be leveraged for higher modulation rates and broader band signals.

Moreover, as the encoding angle varies, the peak-to-peak voltage (Vpp) of the modulation signal decreases, leading to increased overall waveform distortion. This discrepancy in amplitudes between the two states of the TRIS under the same AC signal frequency introduces more harmonic components during OOK modulation, thereby reducing modulation efficiency. Despite these challenges, this platform successfully achieves integrated OOK modulation within the frequency range of DC to 100 MHz and ±60° beam steering, significantly enhancing system simplicity and reducing power consumption.

The proposed TRIS enables wide-angle beam scanning and on-chip OOK modulation. It does this without requiring additional mixer approaches or complex spatiotemporal techniques. This positions it as a promising technology for facilitating sub-terahertz beam steering platforms with OOK modulation and beamforming capabilities. Table 1 presents a summary of various RIS in comparison to key parameters. Our proposed OOK-PB-phase TRIS stands out due to its lowest insertion loss, widest scanning angular ranges, and unique self-OOK modulation capability on the chip. To our knowledge, our proposed TRIS is the first to achieve quasi-continuous beam scanning with OOK modulation without the need for additional modulation components or complex spatiotemporal techniques—a feature not previously reported in existing terahertz or sub-terahertz TRIS technologies.

**Table 1:** Comparison of the correlated RIS for beam steering

Ref.	Freq. (GHz)	RIS type	Element loss^a^ (dB)	Tuning method	Scanning range	Max modulation freq. (MHz)
^21^	23.5	Reflect (Space-Time coding)	3.8	PIN-diode	±45°	1.8
^33^	73	Reflect (Space coding)	6.2	PIN-diode	±70°	N/A
^18^	97	Transmit (Space coding)	11	PIN-diode	30°	N/A
^34^	98	Reflect (Space coding)	9	liquid crystal	35°	N/A
^35^	100	Reflect (Space coding)	8	liquid crystal	±20°	N/A
^17^	300	Transmit (Space coding)	5	CMOS	±30°	N/A
This Work	100	Transmit (Space coding)	2.8	Schottky diode	±60°	100

^a^The element loss is the insertion loss at 0°

## Discussion

Aiming to a new-paradigm wireless communication system operating in the sub-terahertz frequency range, we introduce an OOK-PB-phase reconfigurable intelligent surface that seamlessly integrates beam scanning and OOK modulation on a transmit array. By utilizing PB-phase and Schottky diode switches for 1-bit coding state switching, our proposed TRIS achieves a maximum cross-polarization transmission coefficient of −2.8 dB at 100 GHz, and a −3 dB relative bandwidth of 11%. In beam scanning applications, the TRIS can achieve a maximum scanning angular range of 0° to ±60°. Thanks to its ability to jointly control amplitude and phase, the OOK-PB-phase TRIS enables simultaneous 0°− ± 60° beam scanning of the fundamental frequency and direct OOK modulation of single-tone signals ranging from DC to 100 MHz. Finally, we develop a real-time self-modulated beam point-to-point tracking platform, showcasing up to 100 MHz modulation frequencies on sub-terahertz TRIS. In the future, large-scale diode-based TRIS can be realized through wafer-level lithography processes and surface-mounted technology, potentially enhancing the performance and reliability of TRIS. This novel approach paves the way for advanced terahertz joint sensing and communication (JSAC) systems, secure communication platforms, holographic projectors, and other transformative applications.

## Materials and methods

### Beam scanning measurements

The measurement setup includes a sub-terahertz vector network analyzer (VNA), two collimating lenses, a Field Programmable Gate Array (FPGA) control module, 75~110 GHz T/R modules, and a rotating table. To prevent interference from the surroundings, a sub-terahertz broadband absorbent sponge is placed around the core area of the array during experiments. A normally incident x-polarized wave is collimated onto the TRIS by the lens at a distance of 300 mm, and the deflected y-polarized waves are captured by the receiver lens within a ±70° angular range.

### Construction of an integrated platform combining high-speed beam steering and spatial self-OOK modulation

The platform consists of a transmitter module, the proposed TRIS prototype, a receiver module, and a frequency-adjustable bias module. The transmitter module includes a 12.5 GHz single-tone signal source, a W-band eightfold frequency multiplier, a W-band power amplifier (PA), and a WR-10 (75–110 GHz) horn antenna, capable of delivering a saturated power output of 35 dBm. The receiver module comprises a WR-10 horn antenna, a W-band detector, and an intermediate frequency low-noise amplifier (LNA) to ensure that the received power exceeds the detector’s lower limit.

## Supplementary information

Supplementary Information

Supplementary information (Video)

## Data Availability

The data that supports the plots within this paper and other findings of this study are available from the corresponding author upon reasonable request.
