# Supplementary material for: Sub-terahertz transmissive reconfigurable intelligent surface for integrated beam steering and self-OOK-modulation
Source: Light Sci Appl. 2025 Jan 1;14:13. doi: 10.1038/s41377-024-01690-0 (PMC11688443; doi:10.1038/s41377-024-01690-0)
Supplement: Supplementary file 1 — Supplementary Information [file 41377_2024_1690_MOESM1_ESM.docx]

Supplementary Information for

**Sub-terahertz transmissive reconfigurable intelligent surface for integrated beam steering and self-OOK-modulation**

*Dongfang Shen^1,8^, Feng Lan^1,2,3,8^, Luyang Wang^1^, Tianyang Song^1^, Munan Yang^1^, Tianyu Hu^1^, Yueting Li^1^, Xiaolei Nie^1^, Jiayao Yang^1^, Shixiong Liang^4^, Hongxin Zeng^1^, Hui-Fang Zhang^5^, Pinaki Mazumder^6^, Ziqiang Yang^1,2,3^, Yaxin Zhang^1,2,3^ and Tie Jun Cui^3,7^*

*1 Sichuan THz Communication Technology Engineering Research Center, School of Electronic Science and Engineering, University of Electronic Science and Technology of China, Chengdu 611731, China.*

*2 Yangtze Delta Region Institute (Huzhou), University of Electronic Science and Technology of China, Huzhou 313000, China.*

*3 Zhangjiang Laboratory, Shanghai 201204, China.*

*4 School of Microelectronics, Tianjin University, Tianjin, 300072, China.*

*5 Shenzhen Institute for Advanced Study, University of Electronic Science and Technology of China, Shenzhen 518100, China.*

*6 Department of Electrical Engineering and Computer Science, University of Michigan, Ann Arbor, MI 48109, USA.*

*7 Southeast University, Nanjing 210096, China.*

8 These authors contributed equally: Dongfang Shen, Feng Lan.

*Corresponding authors:*

*Feng Lan (lanfeng@uestc.edu.cn);*

*Yaxin Zhang(*[*zhangyaxin@uestc.edu.cn*](mailto:zhangyaxin@uestc.edu.cn)*);*

*Tie Jun Cui(*[*tjcui@seu.edu.cn*](mailto:tjcui@seu.edu.cn)*);*

**I. The** **meta-atom structure parameters and optimization process**

The 2D diagrams of the top and bottom layers of the meta-atom structure are provided in **Fig. S1**. Specific parameters are listed in **Table S1**.


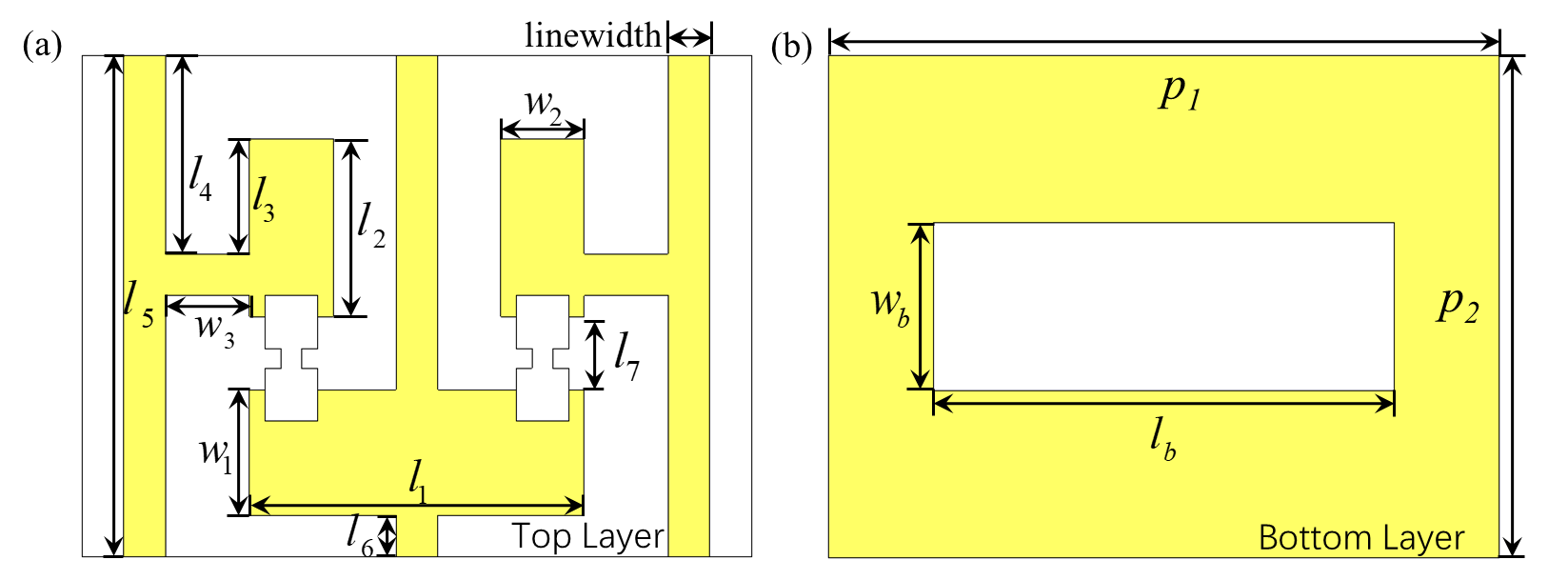


Fig. S1 Schematic of the reconfigurable unit cell (a) Front view (b) Rear view.

The meta-atom structure of TRIS is simulated using CST Studio Suite's frequency domain solver. CST Studio Suite uses tetrahedral mesh to automatically mesh the model. Finally, the minimum boundary length of the mesh is 0.038 μm, the maximum boundary length is 771 μm, the number is 101211, and the average quality is 0.678049. Convergence accuracy is 1×10^-4^ (tetrahedral mesh). Simulation of amplitude and phase response of TRIS’s meta-atom structure based on unit cell boundary conditions.

Based on CST Studio Suite's frequency domain solver, the design process is organized into four main procedures, as depicted in Fig. S2. The design begins with an ideal PB phase shift structure labelled as ①. According to the PB phase principle, a static 180° phase shift can be achieved using two anisotropic L-shaped structures that are 90° rotationally symmetric. Additionally, a U-shaped structure with two Schottky diodes positioned in the gap creates a reconfigurable PB-phase design without the need for bias lines, as indicated by label ②. By alternately switching the two Schottky diodes on and off, a reconfigurable 180° PB-phase shift can be realized, emulating the effect of two 90° rotationally symmetric L-shaped structures. Next, a square-lattice double-split E-shaped structure is developed by integrating three parallel bias wires into the U-shaped structure, forming a building block for a column-wise controlled array layout. Finally, the rectangular-lattice double-split E-shaped structure is obtained by elongating the period *p* and the wire-gap width *w_3_​* of the original square structures, incorporating a bottom rectangular slot to enhance cross-polarization conversion efficiency, as shown by label ④.

The simulated cross-polarized transmission coefficients *T_yx_* for the four structures are presented in Figs. S3 and S4. For both structures ① and ②, the *T_yx_* curves display typical PB-phase characteristics, including consistent non-resonant amplitudes and a smooth broadband 180° phase shift. Due to the absence of a bottom slot to enhance cross-polarization conversion, the maximum *T_yx_* amplitudes are below -6.4 dB. The U-shaped structure, affected by the nonideal switching ratio of the diodes, shows even lower maximum amplitudes of -8.5 dB and a -3 dB bandwidth reduced by 40 GHz. Given that the integration of feeder wires in structure ③ alters the inter-cell and intra-cell coupling, it causes a red shift and bandwidth splitting in the resonance spectrum, as shown in Fig. S4. However, the feeder lines have a negligible impact on the maximum *T_yx_* amplitudes. Compared to the other three structures, structure ④ achieves a significantly improved *T_yx_* amplitude due to the incorporation of a bottom rectangular slot. *T_yx_* amplitudes at 99.5 GHz with -2.5 dB for the two states. The phase difference range of structure ③ is 180° ± 30°. The phase difference range of structure ④ is 180° ± 10°. By optimizing the structure (adding rectangular lattice and bottom slot structure), the phase difference accuracy of *T_yx_* in STATE 0 and STATE 1 has been improved.

The parameter changes of bottom slot structure and the TSM-DS3 dielectric layer thickness have a significant impact on *T_yx_* and require detailed optimization. By adjusting the length and width of the bottom slot structure to optimize *T_yx_* and -3 dB bandwidth, we ultimately determined *w_b_* = 400 μm and *l_b_* = 1100 μm, as shown in Fig. S5(a). As show in Fig. S5(b), the different thicknesses of the TSM-DS3 dielectric layer are calculated. When the thickness increases to 800 μm, resonance is suppressed, and the thickness is not suitable. Compared to thickness = 200 μm, the maximum *T_yx_* of thickness = 500 μm remains the same, with a -3 dB bandwidth increase by 7 GHz. Therefore, 500 μm is determined as the thickness of the TSM-DS3 dielectric layer.

In the sub-terahertz band, the accuracy of PCB technology can be controlled within ±10 μm to ±20 μm [S1, S2]. Compared to the processing requirements of PCB technology (**Table. S2**), the resonant structure and feeder line size of the unit cell need to be optimized. As shown in **Fig. S6**, based on the accuracy of the PCB process, an error analysis (±100 μm) was conducted on the length and width of the bottom slot structure, and the decrease in maximum *T_yx_* did not exceed 1 dB. As shown in **Fig. S7**, the parameters of unit cell structure are overall expanded to 1.1 times and overall reduced to 0.9 times. Compared to the best results, maximum *T_yx_* decreased by 0.2 dB and 0.5 dB, respectively. The errors caused by the PCB process can be accepted.


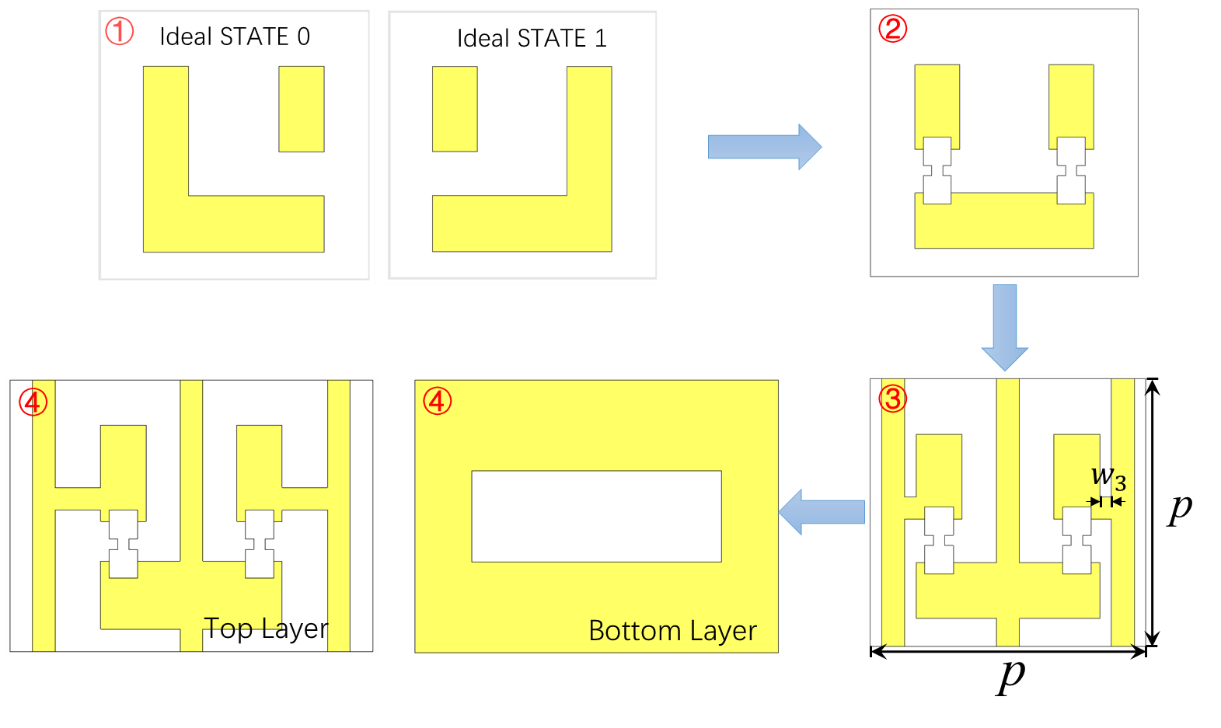


Fig. S2 The design procedure of TRIS’s unit cell structure.


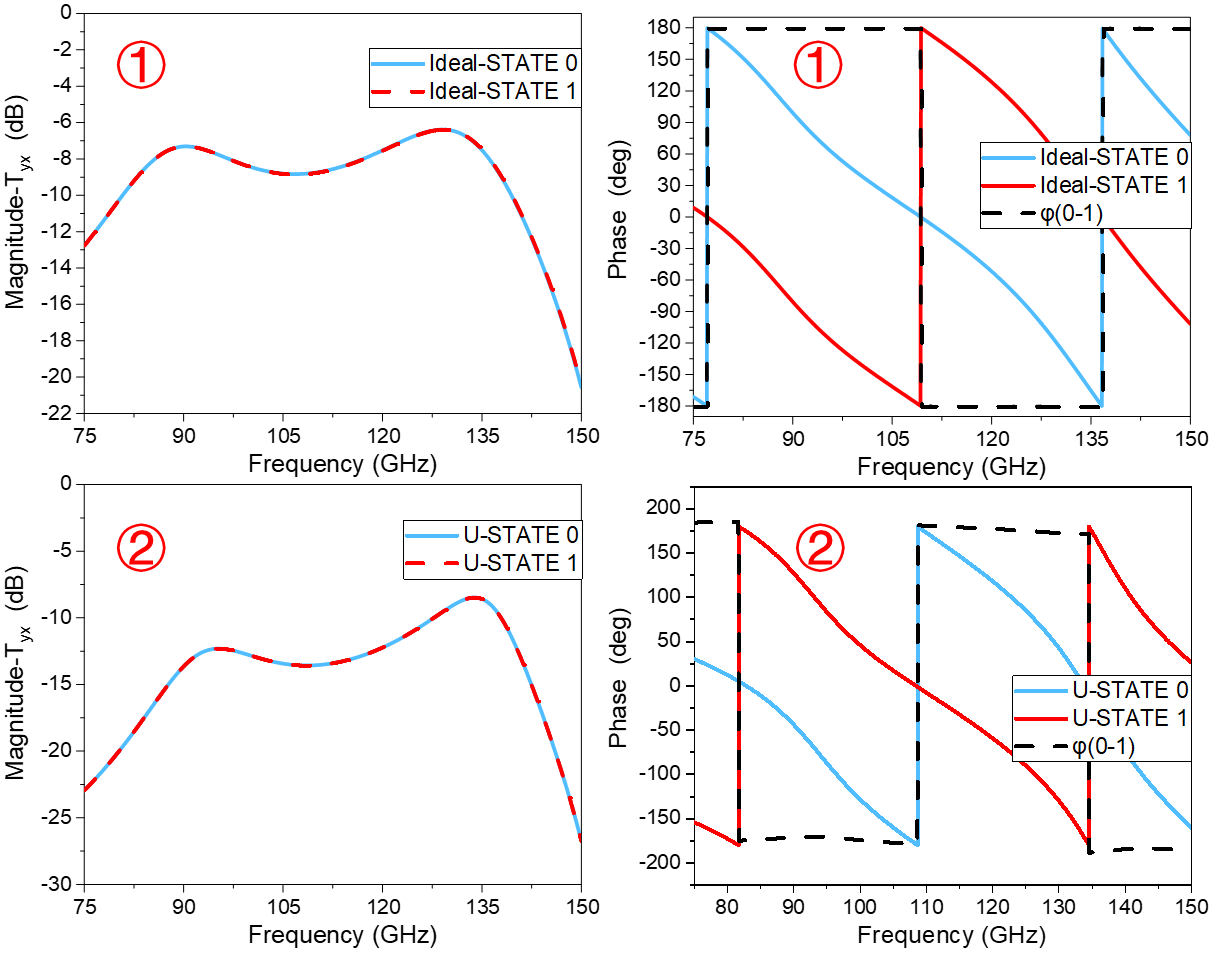


Fig. S3 ①: The *T_yx_* amplitude and phase responses of an ideal PB phase-shift structure for STATE 0 and STATE 1. ②: The *T_yx_* amplitude and phase responses of U-shaped reconfigurable structure for STATE 0 and STATE 1.


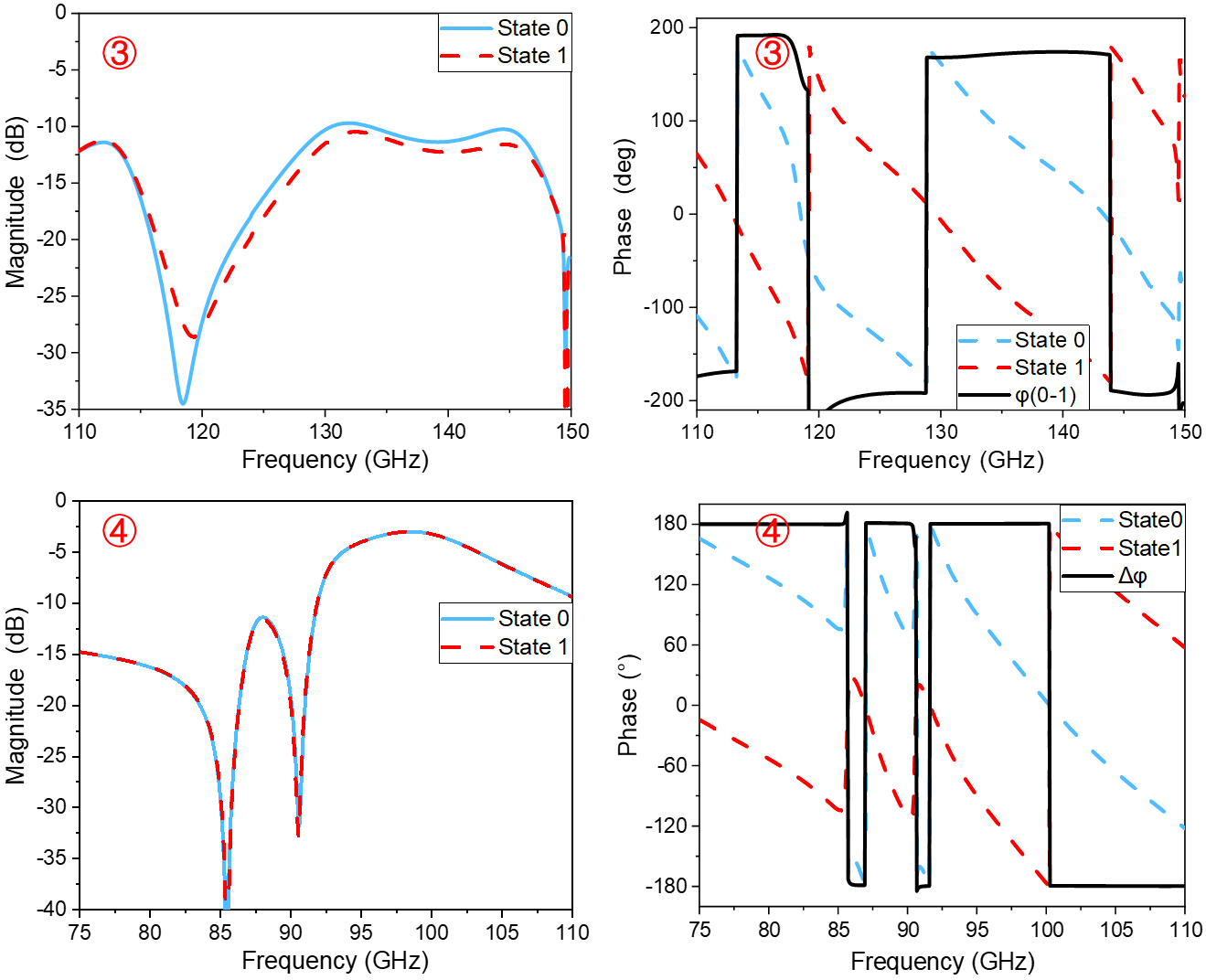


Fig. S4 ③: The *T_yx_* amplitude and phase responses of U-shaped reconfigurable structure with feeder lines for STATE 0 and STATE 1. ④: The *T_yx_* amplitude and phase responses of the rectangular-lattice double-split E-shaped structure for STATE 0 and STATE 1.


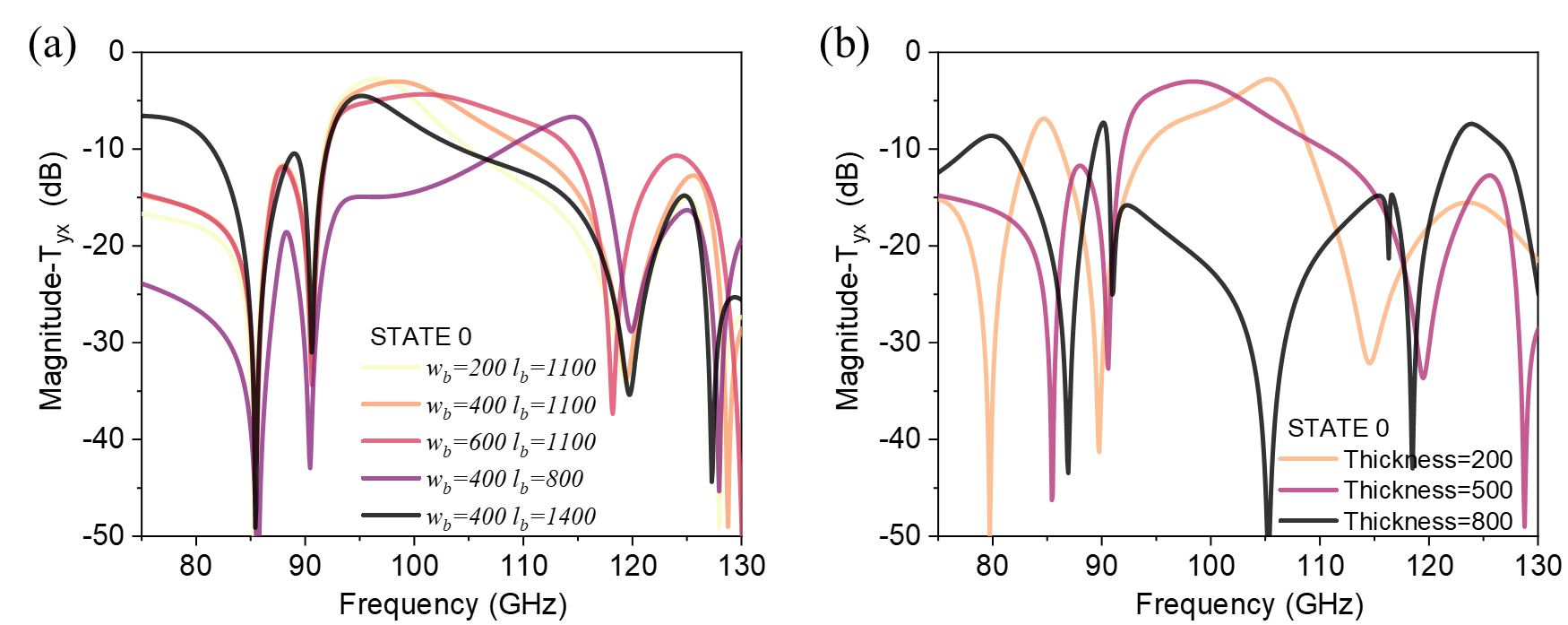


Fig. S5 (a) The influence of bottom slot with different lengths and widths on *T_yx_*_._ (b) The influence of different thickness on *T_yx_*_._

_._
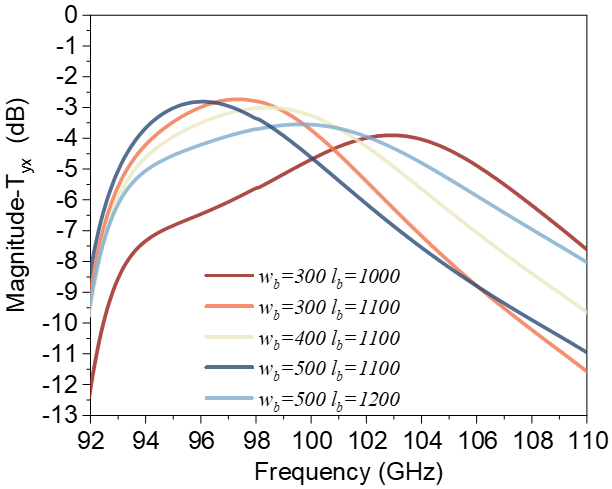


Fig. S6 The influence of bottom slot with different lengths and widths on *T_yx_*_._

Fig. S7 The parameter of unit cell structure error analysis.

**II. Fabry-Perot-like cavity based on multiple interference theories**

From the perspective of transmissive polarization conversion, the high polarization conversion efficiency of the meta-atom structure can be understood through multiple interference theories [S3]. The combination of the bottom slot and the anisotropic structure of the top layer slot creates a Fabry-Perot-like cavity that facilitates multiple reflections and transmissions for improving polarization-conversion efficiency by interference enhancement or cancellations, as illustrated in **Fig. S8**. The top anisotropic structure acts as a polarization converter applying the PB-phase principle, and the bottom rectangular slot acts as a *y*-polarization selector similar to a rectangular waveguide. Specifically, the incident *x*-polarized wave *E_ix_* impinging on the top anisotropic structure is decomposed into the *y*-polarized and *x*-polarized components, with *E_r1_* partially reflected to the air, and the main part of the *x*_1_-pol and *y*_1_-pol transmits into the dielectric spacing. Since the bottom slot filters the *y*-polarized components *E_ty1_* through, the rest of *x*_2_-pol and *y*_2_-pol are reflected to the top layer, interacting with the the top layer again. Then, the above processes are repeated as multiple reflections and transmissions with constructive and destructive interference requirements to cross-polarization and co-polarization, respectively. Finally, the overall cross-polarized transmission coefficient *T_yx_* summed of *E_ty1_, E_ty2_,*…can be significantly enhanced. For a better understanding of the rationale of the bottom slot, the cross-polarized transmission coefficients of *T_yx_* with *x*-polarized incidence and *T_xy_* with *y*-polarized incidence are compared with situations with and without the bottom slot in **Fig. S9**. Compared to the maximum *T_yx_* without a slot structure, the maximum *T_yx_* with slots has increased by 7 dB. The results show that the addition of a bottom slot has a significant effect on the improvement of *T_yx_*.


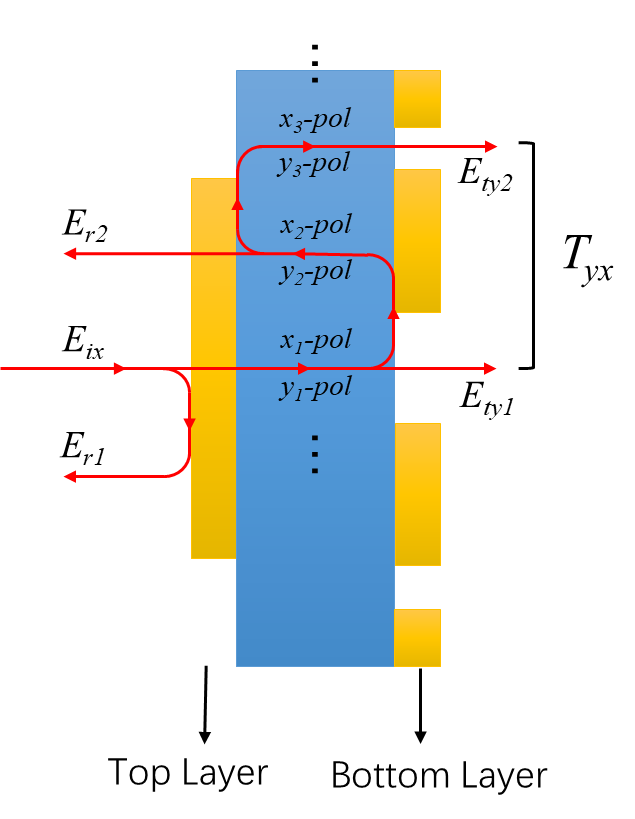


Fig. S8 Schematic of Fabry–Perot-like resonance in transmission mode.


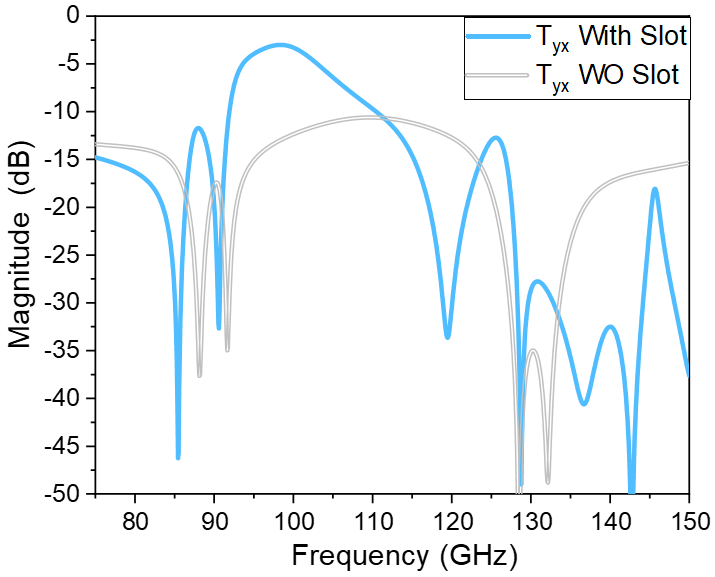


Fig. S9 TRIS amplitude response with bottom slot and without bottom slot.

**III. Link analysis and TRIS amplitude response measurement**

The gain and loss on the Tx to Rx path are shown in **Fig. S10**. Due to Tx/Rx’s the horn antennas and the collimating lenses are completely identical, the standard horn antenna with a gain of 20 dBi and the THz collimating lens with a gain of 6 dBi. The gain of the transmitter and receiver is consistent, i.e. *G_t_* = *G_r_* = 26 dBi. Tx and Rx have been calibrated, so the normalized energy of Tx is 0 dB, i.e. *P_t_* = 0 dB. Based on the gain *G_t_* and aperture area *A_t_* of the transmitting antenna, the efficiency of the transmitting antenna is obtained:

$e_{t}=G_{t}\lambda^{2}/\left( 4\pi A_{t} \right)$ (S1)

By multiplying et with *P_t_*, the total radiated power *P_tt_* of the Tx (Tx, horn antenna and collimating len, as shown in the **Fig. S11**) can be obtained:

$P_{\mathrm{tt}}=e_{t}P_{t}=P_{t}G_{t}\lambda^{2}/\left( 4\pi A_{t} \right)$ (S2)

Therefore, *P_tt_* = *P_t_* = 0 dB. The path loss for 400mm transmission of electromagnetic waves at 100 GHz is approximately -64.2 dB ($20\log_{10} \frac{\lambda}{4\pi r}$). In addition, the spot area of the collimating lens is 507 mm^2^, and the spot area of TRIS is 123 mm^2^. The difference in spot area introduces spillover effects ($L=10\lg\left( {A_{TRIS}}/{A_{Lens}} \right)=6dB$). Finally, the power *P_r_* of TRIS and hollow PCB board were measured by Rx, as shown in Fig. R8. Based on the Friis transmission formula:

$G_{A}=P_{r}-P_{\mathrm{tt}}-G_{r}-20\log_{10} \frac{\lambda}{4\pi r}+L$ (S3)

where *G_A_* is the actual gain of the tested antenna (hollow PCB board or TRIS). The 0° gain *G_A_* of TRIS STATE 0 is approximately 20.4 dBi. The 0° gain *G_A_* of hollow PCB board is approximately 23.2 dBi. Their areas are equal, and subtracting them yields the *T_yx_* of STATE 1 is -2.8 dB. Similarly, the *T_yx_* of STATE 1 is -4 dB.

**
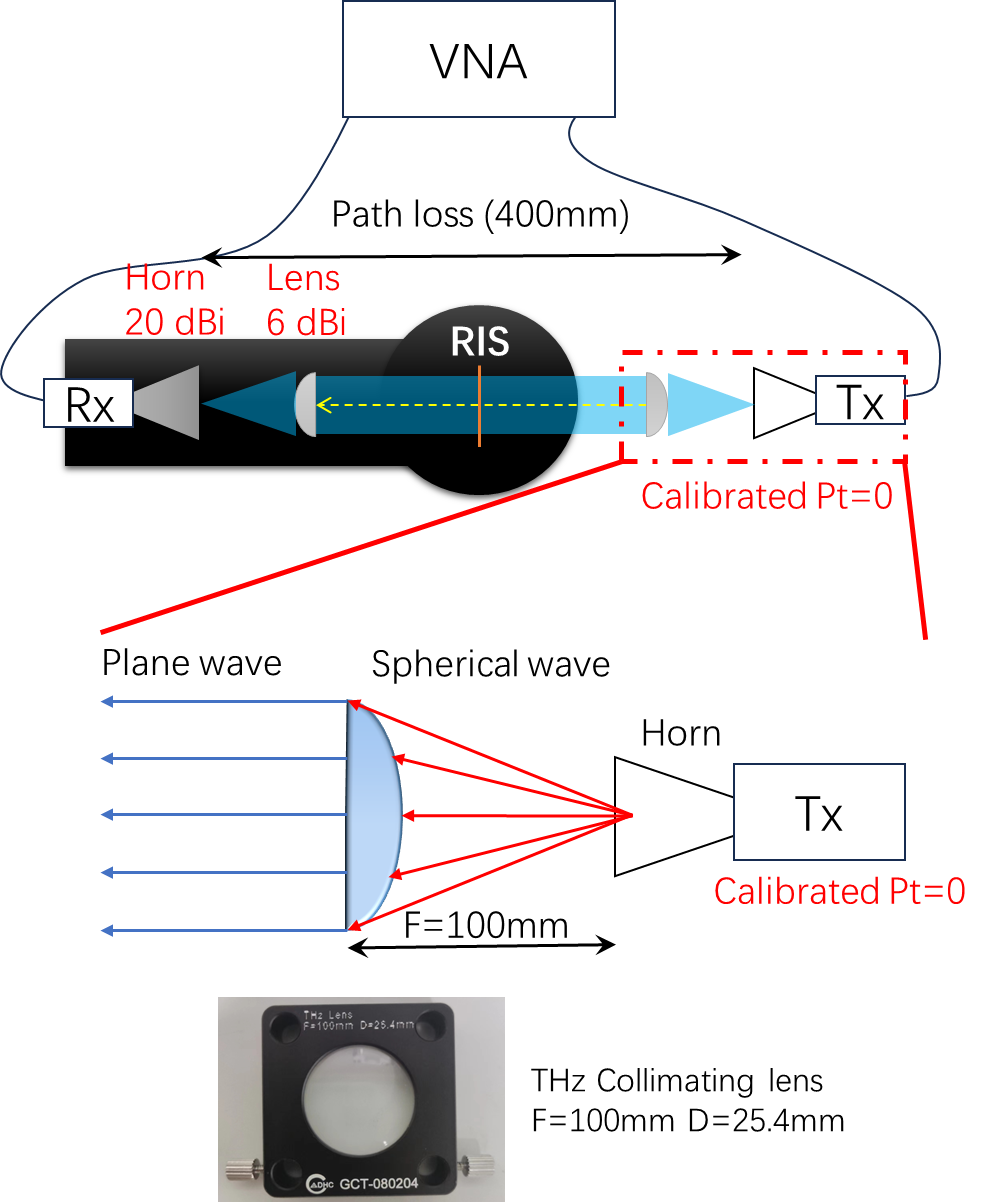
**

Fig. S10 Schematic of Link analysis from Tx to Rx.

**
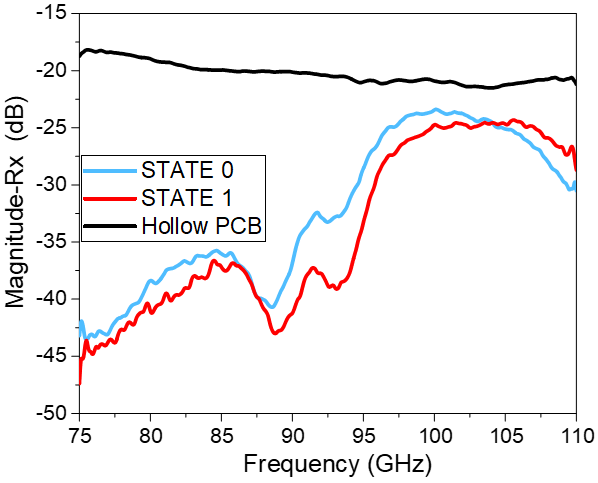
**

Fig. S11 The actual magnitude of TRIS and hollow PCB board measured at Rx

Table. S1 The parameter of the meta-atom structure.

| Parameter | Value (unit: μm) | Parameter | Value (unit: μm) |
| --- | --- | --- | --- |
| *l_1_* | 800 | *l_b_* | 1100 |
| *w_1_* | 300 | *w_b_* | 400 |
| *l_2_* | 425 | *l_4_* | 475 |
| *w_2_* | 200 | *l_5_* | 1200 |
| *l_3_* | 275 | *l_6_* | 100 |
| *w_3_* | 200 | *l_7_* | 175 |
| *p_1_* | 1600 | *p_2_* | 1200 |

Table. S2 The PCB process limitations.

| The PCB process | Value (unit: μm) |
| --- | --- |
| Minimum metal linewidth | 100 |
| Minimum metal spacing (metal to metal) | 76.2 |
| Minimum metal thickness | 18 (0.5oz) |
| Minimum TSM-DS3 dielectric layer thickness | 127 |
| Dimensional error | ±10 |

Table. S3 Link analysis of Tx to Rx (100 GHz).

| Link Analysis (100 GHz) | Parameter |
| --- | --- |
| P_tt_ (Calibrated) | 0dB |
| Path Loss (400 mm) | -64.2 dB |
| Loss (Energy spillover) | 6 dB |
| G_r_ | 26 dBi |
| P_r_ (Hollow PCB board) | -21 dB |
| P_r_ (STATE 0 of TRIS) | -23.8 dB |
| P_r_ (STATE 1 of TRIS) | -25 dB |
| TRIS Gain (0°) | 20.4 dBi |

**Reference:**

[S1] Medrar, Kossaila, et al. "H-band substrate-integrated discrete-lens antenna for high data rate communication systems." IEEE Transactions on Antennas and Propagation 69,7 (2020) DOI: 10.1109/TAP.2020.3044382.

[S2] Shi, Suyang, et al. "Wideband polarization rotation transmitarray using arrow-shaped FSS at W-band." IEEE Transactions on Antennas and Propagation 70,7 (2022) DOI: 10.1109/TAP.2022.3140309.

[S3] Nathaniel K. Grady et al., Terahertz Metamaterials for Linear Polarization Conversionand Anomalous Refraction.Science340,1304-1307(2013). DOI :10.1126/science.1235399.
